# Functional Cardiomyocytes Derived from Isl1 Cardiac Progenitors via Bmp4 Stimulation

**DOI:** 10.1371/journal.pone.0110752

**Published:** 2014-12-18

**Authors:** Esra Cagavi, Oscar Bartulos, Carol Y. Suh, Baonan Sun, Zhichao Yue, Zhengxin Jiang, Lixia Yue, Yibing Qyang

**Affiliations:** 1 Yale Cardiovascular Research Center, Section of Cardiovascular Medicine, Department of Internal Medicine, Yale Stem Cell Center, Yale School of Medicine, Yale University, New Haven, CT, United States of America; 2 Department of Medical Biology, School of Medicine, Istanbul Medipol University, Istanbul, Turkey; 3 Department of Genetics, Yale School of Medicine, Yale University, New Haven, CT, United States of America; 4 Department of Cell Biology, University of Connecticut Health Center, Farmington, CT, United States of America; 5 Department of Pathology, Yale School of Medicine, New Haven, CT, United States of America; 6 Vascular Biology and Therapeutics Program, Yale University School of Medicine, New Haven, CT, United States of America; Centro Cardiologico Monzino, Italy

## Abstract

As heart failure due to myocardial infarction remains a leading cause of morbidity worldwide, cell-based cardiac regenerative therapy using cardiac progenitor cells (CPCs) could provide a potential treatment for the repair of injured myocardium. As adult CPCs may have limitations regarding tissue accessibility and proliferative ability, CPCs derived from embryonic stem cells (ESCs) could serve as an unlimited source of cells with high proliferative ability. As one of the CPCs that can be derived from embryonic stem cells, Isl1 expressing cardiac progenitor cells (Isl1-CPCs) may serve as a valuable source of cells for cardiac repair due to their high cardiac differentiation potential and authentic cardiac origin. In order to generate an unlimited number of Isl1-CPCs, we used a previously established an ESC line that allows for isolation of Isl1-CPCs by green fluorescent protein (GFP) expression that is directed by the *mef2c* gene, specifically expressed in the Isl1 domain of the anterior heart field. To improve the efficiency of cardiac differentiation of Isl1-CPCs, we studied the role of Bmp4 in cardiogenesis of Isl1-CPCs. We show an inductive role of Bmp directly on cardiac progenitors and its enhancement on early cardiac differentiation of CPCs. Upon induction of Bmp4 to Isl1-CPCs during differentiation, the cTnT+ cardiomyocyte population was enhanced 2.8±0.4 fold for Bmp4 treated CPC cultures compared to that detected for vehicle treated cultures. Both Bmp4 treated and untreated cardiomyocytes exhibit proper electrophysiological and calcium signaling properties. In addition, we observed a significant increase in Tbx5 and Tbx20 expression in differentiation cultures treated with Bmp4 compared to the untreated control, suggesting a link between Bmp4 and Tbx genes which may contribute to the enhanced cardiac differentiation in Bmp4 treated cultures. Collectively these findings suggest a cardiomyogenic role for Bmp4 directly on a pure population of Isl1 expressing cardiac progenitors, which could lead to enhancement of cardiac differentiation and engraftment, holding a significant therapeutic value for cardiac repair in the future.

## Introduction

Heart failure caused by myocardial infarction remains a leading cause of morbidity and mortality in the developed world [1]. Current therapies can slow the progression of heart failure, but there are limited options to repair or reverse injury to the myocardium. Many are exploring the potential of injecting stem cells into the heart to generate new myocardium to develop cell-based cardiac regenerative therapies. Various cell types have been tested for cardiac regeneration and some have shown improvement in cardiac function. However, the differentiation and long-term engraftment of the injected stem cells has been challenging. In addition, some of the early human clinical trials have shown promising, but limited differentiation capacity and variable improvements in heart function after myocardial injury [1].

Cardiac progenitor cells (CPCs) have become a potential source for cell therapy due to their cardiovascular differentiation potential. Over the last decade, various CPC populations have been suggested based on expression of certain transcription factors or cell surface markers; such as Islet-1 (Isl1+) [2], KDR+ (Flk1) [3], cKit+ lineage- [4], cardiospheres [5], [6], side population cells [7] and Tbx18 epicardial stem cells [8]. With the goal of regenerating cardiomyocytes, smooth muscle cells, and endothelial cells, CPCs can potentially be a source of differentiating cells upon injection into an injured myocardium, thus regenerating the damaged tissue and replacing it with new tissue. While adult cardiac progenitor cells can provide a valuable cell source for cell-based therapy, their derivation and isolation from adult tissue may be limited due to tissue accessibility and they may have limited proliferative ability, depending on the age of the tissue donor. Additionally, patients with cardiovascular diseases or aged patients may have functionally compromised adult stem cells and may experience decline in therapeutic function.

On the other hand, investigators have developed ways to generate cardiac progenitor cells from pluripotent stem cells. Cardiac progenitor cells derived from embryonic or induced pluripotent stem cells can serve as a potential source of cells for cell therapy due to their capability for unlimited self-renewal and differentiation capacity. However, it is still debated whether some of these CPC populations can be classified as true resident heart progenitors and their identification criteria are unclear. Of these populations, the progenitors shown to be of cardiac origin were based on developmental studies of those expressing the LIM-homeodomain protein Isl1. Isl1 cardiac progenitor cells are a more restricted subpopulation of progenitors that are derived from the second heart field (SHF; or anterior heart field: AHF) during embryonic development. Elegant lineage tracing experiments provide evidence that, Isl1-expressing cardiac progenitor cells (Isl1-CPCs) contribute to more than two thirds of the heart and give rise to the three main cell types in the heart: cardiomyocytes, smooth muscle cells and endothelial cells [2], [9]–[11], all of which are required for cardiac repair *in vivo*. With demonstrated cardiac origin and high cardiac differentiation potential, Isl1-CPCs are a potential source for cell based cardiac repair.

Several laboratories have isolated and characterized Isl1-CPCs from differentiation of embryonic stem cells (ESCs) [10], [12]–[14]. The Cre recombinase gene controlled by the *ISL1* promoter and a Dsred or yellow fluorescent protein (YFP) reporter was used for the isolation and fate-mapping of *ISL1^+^* cardiovascular progenitor cells (ICPCs) [12], [13]. However, the *Dsred^+^* or *YFP^+^* cell populations may represent a mixture of both ICPCs and differentiated progenies from ICPCs as well as extracardiac populations, since *ISL1* is expressed in other tissues besides heart tissue [15]–[17]. In addition to transgenic methods, Nsair et al. 2012 sought to identify specific cell surface markers to identify and isolate Isl1+ CPCs. Although they were able to identify Flt1+/Flt4+ cells were the best to identify Isl1+ CPCs, the biology of the Flt1+/Flt4+ Isl1 CPCs needs to be further investigated, such as the lineage tracing or fate mapping of these identified cells [14].

In order to generate a pure population of cardiac progenitor cells, Qyang et al. 2007 established an ESC line in which green fluorescent protein (GFP) is directed by a fragment of the *mef2c* gene that is specifically expressed within the *Isl1* domain of the anterior heart field (AHF) [18], [19]. Upon isolation of the GFP+ cells, the GFP+ Isl1 CPCs spontaneously differentiate into cardiomyocytes. However, the cardiac differentiation efficiency remains low and is largely unknown, while the functions of these differentiated cardiomyocytes are not well characterized. In order to use Isl1 CPCs as a potential source for cell therapy, highly efficient cardiac differentiation of Isl1-CPCs needs to be achieved in order to harness their cardiac repair potential to generate a sufficient number of cells to recover the massive loss of cardiomyocytes upon injury. Thus, defining ways to improve the efficiency of differentiation is a prerequisite to acquire large numbers of pure progenitors and differentiated functional progeny. One of the main challenges of using ESC-derived cardiovascular cells is the potential formation of teratoma. The risk of teratoma formation should be minimal with Isl1-CPCs since i) these CPCs are highly committed cardiovascular cells and ii) we can further purify them by cell sorting. Through monitoring of genomic integrity by karyotyping and other genomic tools, Isl1-CPCs may provide a promising supply of cells for cardiac repair and regeneration.

Bone morphogenetic proteins (Bmps) have been suggested to promote cardiac differentiation in ESC cultures. Bmps belong to the TGF-beta family secreted factors and have been developmentally implicated in driving cardiac differentiation both *in vitro* and *in vivo* models [20], [21]. Among a dozen Bmps, the activating Bmp4 ligand is expressed at the appropriate developmental stages in heart muscle development [21]. Moreover, myocardial differentiation was delayed in Bmp2 and Bmp4 conditional mutant embryos [22]. A micro-RNA mediated link has been discovered through which Bmp signaling regulates cardiac differentiation from cardiac progenitors [22]. Furthermore, the role of Bmp signaling in Isl1 progenitors has been addressed by using an Isl1-Cre based knock-out strategy for Bmpr1a, a Type I Bmp receptor [23]. Although, this study suggested a requirement for Bmp signaling in Isl1-expressing progenitors, these genetic lineage experiments also tracked neural crest derived Isl1 derivatives, which are not restricted to second heart field, potentially complicating the interpretation of their findings [17], [23]. Later studies used a more stringent genetic strategy, to specifically track and isolate authentic cardiac lineages expressing Isl1, by following the expression of the GFP protein that is transcriptionally regulated by Isl1 and Gata4 (AHF-GFP) [17], [18], but the role of Bmp4 in these restricted and specific Isl1-CPC populations has not been established yet.

In this study, we aimed to investigate the critical challenge of improving cardiac differentiation of pure, homogeneous cardiovascular progenitors. We characterized specifically Isl1 and Gata4 expressing CPC-derived cardiomyocytes and the role of Bmp4 on cardiogenesis. We demonstrate an inductive role of Bmp directly on cardiac progenitors and its enhancement on early cardiac differentiation of CPCs. Expression of Tbx5 and Tbx20 is enhanced upon the induction of Bmp4, which suggests an activation of early cardiogenic program/genes by Bmp4 in Isl1-CPCs. Bmp4-induced Isl1-derived cardiomyocytes appear to express similar action potentials and calcium channeling properties as exhibited by uninduced cardiomyocytes. This study is the first to show that Bmp4 induces cardiomyogenesis in a pure population of Isl1 expressing cardiac progenitors. Collectively these findings suggest a cardiomyogenic role for Bmp4 directly on cardiac progenitors. Based on our findings, application of Bmp4 for cardiac repair using regenerative medicine strategies could lead to enhancement of cardiac differentiation after implantation, holding a significant therapeutic value for cardiac repair in the future.

## Materials and Methods

### Cell culture

AHF-GFP mouse embryonic stem cell line, which was previously derived from AHF-GFP transgenic mouse line, was used [18]. This transgenic line allows for Isl1-CPC detection and isolation from cardiac differentiation cultures by GFP expression, which is linked to specific mef2c gene promoter fragment, a direct target of Isl1 at the anterior heart field (AHF). AHF-GFP mESC were cultured on irradiated-MEF feeder cells in Dulbecco's modified Eagle's medium (DMEM, Invitrogen) supplemented with 15% Knock-out serum replacement (KO-SR, Invitrogen), LIF-conditioned media at 1∶500 dilution (LIF conditioned media was collected from Chinese hamster ovary (CHO) cells stably expressing Leukemia Inhibitory Factor gene), 0.1 mM non-essential amino acid (MEM-NEAA, Invitrogen), 2 mM L-glutamine (Invitrogen), 1 mM sodium pyruvate (Invitrogen), 1% penicillin and streptomycin (Invitrogen) and 0.1 mM 2-mercaptoethanol (Sigma) as described previously.

### In vitro differentiation

AHF-GFP ESCs were cultured to reach 70–80% confluency. Cultures were then dissociated with 0.25% trypsin-EDTA (Invitrogen). Dissociated ESCs were cultured on gelatin-coated tissue culture dishes in order to adapt to feeder-free conditions. During this adaptation stage, ESCs were grown in Iscove's modified Dulbecco's medium (IMDM, Invitrogen) supplemented with 15% KO-SR, LIF-conditioned medium, 2 mM L-glutamine, 1% penicillin and streptomycin and 0.1 mM 1-thioglycerol (Sigma). After 2 days in feeder-free culture, mESC colonies were dissociated and hanging drops containing 500–600 ESCs in 15 µl differentiation medium were prepared. Embryoid bodies (EBs) formed within 2 days of hanging drop cultures in differentiation medium containing IMDM, 15% fetal bovine serum (Gemini), 50 µg/ml ascorbic acid (Sigma), 0.1 mM 1-thioglycerol (Sigma) and 2 mM L-glutamine.

For cardiomyocyte differentiation, 2×10^4^ FACS-purified Isl1-CPCs were seeded on one well of Fibronectin (Sigma) coated 384-well tissue culture plates (Grenier). For counting analysis, 12×10^4^ Isl1-CPCs were seeded on 16-well chamber slides (Lab-Tek). 6 hours after plating, recombinant mouse Bmp4 protein (25 ng/ml, R&D systems) or vehicle control was added to cultures. The cardiac differentiation was monitored for 6 days and cells were harvested at designated time points for mRNA analysis and immunostaining.

For smooth muscle cell differentiation, the Isl1-CPCs were cultured under the same conditions as described above for cardiomyocyte differentiation. For endothelial cell differentiation, FACS-purified Isl1-CPCs were grown on endothelial basal medium-2 (EBM-2, Lonza), containing 2% FBS.

### Immunocytochemistry

FACS-sorted GFP+ CPCs from EB day 5.5 differentiation cultures were plated and treated with Bmp4 as described above. 6 days following treatment, cultures were briefly fixed in 4% PFA in Phosphate Buffered Saline (PBS, Gibco) for 10 min at RT and permeabilized for 10 min with PBS and 0.1% TritonX (PBST, Bio-Rad). Cells were blocked with PBST containing 10% normal goat serum (NGS, Invitrogen) for 30 min. Fixed and blocked cells were incubated with antibodies to cardiac Troponin T (cTnT, 1∶1000, Thermo Fisher) in order detect cardiomyocytes; or with antibodies to alpha-smooth muscle actin (SMA, 1∶250, Abcam) in order detect smooth muscle cells; or with antibodies to PECAM-1 (CD31, 1∶200, Abcam) in order to detect endothelial cells, for 60 min at room temperature or overnight at 4°C. Next, cells were immunolabelled with fluorophore-conjugated mouse or rabbit-specific secondary antibodies (AlexaFluor-488 or AlexaFluor-568) (1∶1000, Invitrogen). Nuclei were stained by using Bisbenzimide H 33258 dye (Hoechst, 1∶1000, Sigma). Fluorescence was visualized with Nikon fluorescence microscope under 10-100X magnification.

### Fluorescence activated cell sorting (FACS) analysis

Following 6 days of cardiac differentiation, cultures were enzymatically digested by using collagenase A (6.67 mg/ml, Roche) and collagenase B (6.67 mg/ml, Roche) in differentiation media for 30 min. In order to obtain a uniform single cell suspension, cell clumps were digested 5 min with Accutase (Invitrogen). For FACS-sorting live Isl1-CPC, single cell suspension was filtered through a sterile cell strainer (BD biosciences). GFP- and GFP+ cell populations were sorted into 1∶1 FBS and differentiation media by using Mo-Flo FACS-sorter (Beckman Coulter) at cell sorter core facility at Yale University.

For FACS analysis of cardiomyocytes, single cell suspension was prepared as described above and fixed with 2% paraformaldehyde (PFA) in PBS. Next, fixed cells were blocked and permeabilized in PBST containing 10% Normal Goat Serum for 60 min. Samples were incubated with primary antibody for cTnT in PBST for 60 min at RT, followed by Alexa fluor-647 secondary antibody (Invitrogen) for mouse for 30 min at RT. The labelled cell population was analysed by using LSRII (BD biosciences) and Flow-Jo software (Treestar).

### Cytospin of Isl1-CPCs

Isl1-CPCs were isolated by FACS from EB5.5 culture and processed for cytospin. Cytospins were performed with fifty to a hundred thousand FACS isolated cells (GFP+) at 500 rpm for 3 minutes. Samples were air dried and fixed with 4% paraformaldehyde. Immunofluorescence stainings were performed with mouse anti-Isl1 antibody (Hybridoma Bank) and rabbit anti-alpha smooth muscle actin (SMA from Abcam), cardiac troponin T (cTnT from Abcam) or CD31 (Abcam). For double staining SMA/cTnT, a mouse anti-cTnT antibody (Thermo Scientific) was used. In all the stainings, goat anti mouse-A568 and goat anti rabbit-A647 antibodies (both from Life Technologies) were used as secondary antibodies.

### Action potential recordings

AHF-GFP differentiation cultures at EB day 5.5 were dissociated and FACS-sorted as described above. GFP+ cardiac progenitors were plated onto fibronectin-coated cover slips and cultured for 6 days in the absence or presence of Bmp4 (25 ng/ml, R&D systems) under whole-cell current clamp mode using an Axon Axopatch 200B amplifier and pclamp8 software (Molecular Devices, USA) at RT. Patch pipettes were pulled from borosilicate glass tubes to give a resistance about 3 MΩ when filled with pipette solution. Spontaneous action potential firings of the cells were recorded under continuous recording mode. Data were low-pass filtered at 1 kHz and digitized at a rate of 10 kHz. The bath solution contained 140 mM NaCl, 3 mM KCl, 1 mM CaCl_2_, 1 mM MgCl_2_, 10 mM HEPES, pH7.3 and the pipette solution was 145 mM KCl, 5 mM NaCl, 2 mM CaCl_2_, 2 mM MgCl_2_, 3 mM MgATP, 4 mM EGTA, and 10 mM HEPES, pH7.3.

### Calcium transient measurements

Freshly FACS-sorted GFP+ CPCs from EB day 5.5 differentiation cultures were plated at a density of 3×10^5^/cm^2^ on fibronectin-coated glass cover slips and cultured for 6 days in the absence or presence of Bmp4 (25 ng/ml, R&D systems). Cells were loaded with cell permeant Ca^2+^ indicator dye 10 µmol/L Fura-2 acetoxymethyl ester (Molecular Probe) and 0.1% pluronic F-127 (Sigma) in Tyrode solution containing 2.5 mM Ca^2+^ for 30 minutes at 37°C. Fluorescence intensities at 510 nm with 340 nm and 380 nm excitation were collected at a rate of 1 Hz using CoolSNAP HQ2 (Photometrics) and data were analyzed using NIS-Elements (Nikon). Cytosolic Ca^2+^ of spontaneous beating cardiomyocyte was measured by ratio of fluorescence intensity at 340 nm and 380 nm (F340/F380).

### Quantitative real-time PCR

Total mRNA was collected from FACS-sorted populations and differentiation cultures by using micro or mini RNeasy Kit (Qiagen) or Trizol (Life Technologies). mRNA quantities were measured and cDNA synthesis was performed for 500 ng of mRNA by using iScript cDNA synthesis kit (Bio Rad). Quantitative RT-PCR analysis was performed using iQ SYBR green supermix (Bio Rad) on a CFX96 Touch real-time PCR detection system (Bio Rad). The qRT-PCR data was analyzed by using the CFX-Manager software (Bio Rad), and house keeping gene Gapdh was used for normalization. Isl1 qPCR primers and qRT-PCR reaction details were described previously (Qyang 2007). The qRTPCR primer sets used for PCR reaction are as follows: Gata4-F “gacttctcagaaggcagagag”; Gata4-R “ccatggagcttcatgtagagg”; Nkx2.5-F “cagtggagctggacaaagcc”; Nkx2.5-R “tagcgacggttctggaacca”; Tbx5-F “tgactggccttaatcccaaa”; Tbx5-R “acaagttgtcgcatccagtg”; Tbx20 “gtgcacatcataaagaagaaagacc”; Tbx20-R “aaacggattgctgtctattttcagc”; Cacna1c-F “agcaagaaccactgcggat”; Cacna1c-R “ gaagaaatgcagcaacagcc”; Cacna1d-F “catcccattccctgaagatg”; Cacna1d-R “ggatgcagcaacagtccata”; Ryr2-F “cgaggatgagatccagttcc”; Ryr2-R “caaatccttctgctgccaag”; Serca2-F “gggcaaagtgtatcgacagg”; Serca2-R “tcagcaggaactttgtcacc”; Ncx1-F “gactttgaggacacctgtgg”; Ncx1-R “tcactcatctccaccagacg”; Gapdh-F “ggtgctgagtatgtcgtgga”; Gapdh-R “cggagatgatgacccttttg”.

### Statistical analysis

The findings are represented as the mean ± standard error. The results were analysed by Student's t-test and significance was defined as P values <0.05.

## Results

### Bmp4 enhances cardiomyogenesis in mESC-derived Isl1+ cardiac progenitors

In order to isolate Isl1-CPCs in large quantities for further characterization, we have used an established ESC line-derived from a transgenic mouse strain (AHF-GFP) in which GFP expression is specifically directed by a promoter containing the minimal enhancer sequence from Isl-1 target gene, MEF2c, which has been shown to be expressed within the Isl1 domain of the anterior heart field (AHF) [18], [19]. Although Isl1-CPCs derived from this transgenic ESC line could differentiate into cardiomyocytes, the characteristics of these cardiomyocytes remains largely unknown and the cardiac differentiation efficacy of these Isl1-CPCs needs to be improved. Cardiac differentiation was induced by aggregation of 500–600 mouse ESCs into embryoid bodies (EBs) using our recently established high density EB differentiation approach [24], [25]. At EB day 5.5, Isl1-CPCs were FACS-sorted based on the distinct GFP expression resulting in an average of 11.6% (8.2% to 12.8%) GFP+ CPC population (S1A Fig.). To validate the CPC identity of the GFP+ cells, we measured the mRNA expression of cardiac progenitor marker genes *isl1*, *gata4* and *nkx2.5* and showed that mRNA expression of these genes were significantly enriched in GFP+ cells compared to GFP- population (S1B Fig.). Double staining for progenitor marker Isl1 and cardiomyocyte marker cardiac troponin T (cTnT), smooth muscle cell marker smooth muscle α-actin (SMA) and endothelial cell marker CD31 revealed that FACS-purified GFP+ cells were highly enriched CPCs (83.8%, S1C-E Fig.). Interestingly, consistent with the studies shown by Bu et al. and Musunuru et al., a low percentage of these CPCs co-expressed lineage markers, suggesting that they have committed to lineage-restricted progenitors [13], [26].

To confirm the cardiovascular differentiation potential of Isl1-CPCs *in vitro*, freshly sorted GFP+ Isl1-CPC were cultured for 6 days and immunostained for cTnT, SMA and CD31 (S2A-D Fig.). Cardiomyocytes, smooth muscle cells and endothelial cell lineages were derived from purified Isl1-CPCs, evident by specific staining for lineage specific markers. Our results reiterates that, AHF-GFP ESC line is a reliable system for generation of large quantities of Isl1-CPCs, which have strong cardiovascular origin by gene expression profile as well as developmental potential to generate all three major cell types *in vitro* which constitutes the majority of the heart.

**Figure 1:**
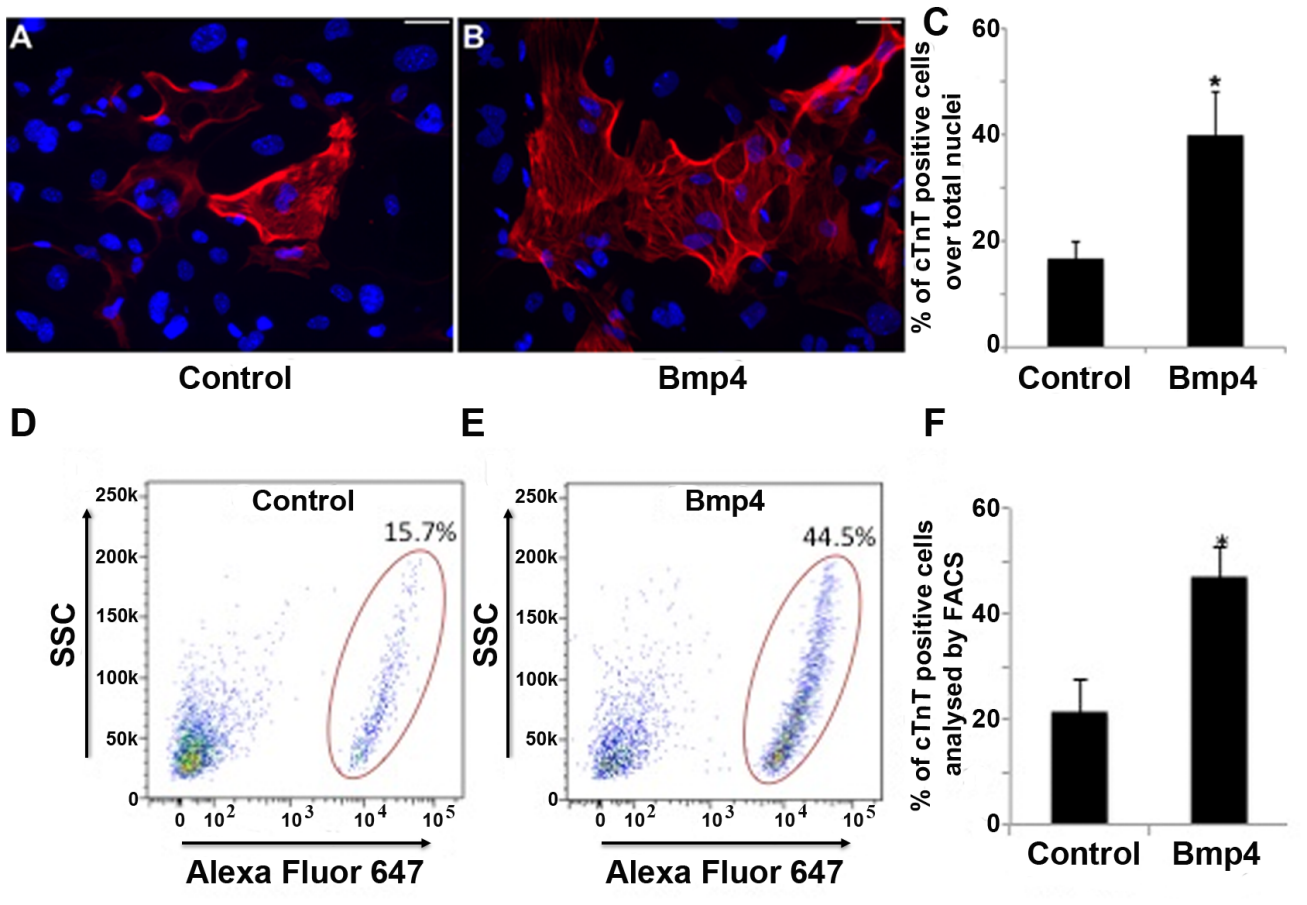
BMP signaling enhances Isl1-CPC cardiac differentiation. Freshly sorted EB day 5.5 GFP^+^ Isl1-CPC were treated with control (A, D) or 25 ng/ml Bmp4 (B, E) for 6 days, followed by immunostaining with antibodies against cardiomyocyte-specific protein cTnT (red in A and B) or labelled with cTnT and Alexa fluor 647 for FACS analysis (D, E). Nuclei: blue (Hoechst). (C) Quantification of A and B (over 2000 cells counted from 3 independent experiments). (F) Quantification of D and E (20000 cells FACS analyzed for each of 3 independent experiments). Scale bar: 100 µm; *statistical significance *p<0.05*, Bmp4 –treated cultures vs. control.

Based on previous reports suggesting a role for BMP signaling in heart muscle development and cardiac differentiation, we hypothesized that modulation of cardiogenic pathway with Bmp4, an activating BMP ligand, may enhance the cardiac differentiation of Isl1-CPCs. To determine whether Bmp4 signaling modulates cardiac differentiation of Isl1-CPC, we cultured FACS-purified Isl1-CPCs in the presence of Bmp4 for 6 days and measured cardiomyogenesis by immunostaining (Fig. 1A-C) or FACS-analysis (Fig. 1D-F) for cardiomyocyte-specific marker cTnT. Quantification of the results of four independent experiments revealed that the cTnT+ cardiomyocyte population was enhanced 2.4±0.3 fold (Fig. 1C; manual counting) and 2.8±0.4 fold (Fig. 1F; FACS analysis) for Bmp4 treated CPC cultures compared to that detected for vehicle treated cultures. In addition, immunofluorescence staining of cTnT showed sarcomeric striated structure (S3 Fig.). These results indicate that Bmp4 induces cardiomyocyte differentiation of FACS purified Isl1-CPCs.

### Electrophysiological characterization of Isl1-CPC-derived cardiomyocytes reveal all three types of cardiomyocytes

**Figure 2:**
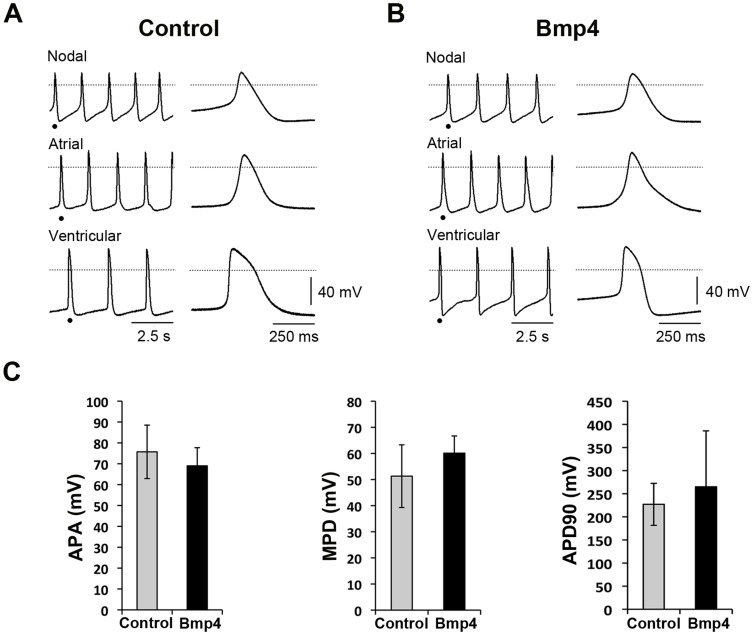
Isl1-CPC derived cultures have cardiomyocyte-like electrophysiological properties. Action potentials of differentiated Isl1-CPCs from control (A) or Bmp4-treated cultures (B) were recorded after 6 days in culture under whole-cell current clamp mode. (A, B) Representative action potentials demonstrate nodal-like, atrial-like and ventricular-like cardiomyocytes in the absence and presence of Bmp4. (C) Action potential amplitude (APA), action potential duration at the 90% of repolarization (APD_90_) and maximum diastolic potential (MDP) for ventricular-like cardiomyocytes were quantified and depicted as bar graph for both control and Bmp4-treated cultures. A total of 29 cardiomyocytes derived from Isl1-CPCs was analyzed from 4 independent experiments. There were no significant differences for APA, MDP and APD_90_ between control and Bmp4 treated samples. The details for AP measurement setting are under Materials and Methods.

We have observed widespread spontaneous beating cardiomyocyte clusters/foci (See S1 Movie and S2 Movie) derived from FACS-purified Isl1-CPCs within 6 days of culture. Quantification of beating clusters/foci revealed that Bmp4 treatment appeared to enhance the number of beating clusters/foci compared to control treatment (S4 Fig.). Previous studies demonstrated diverse action potential (AP) morphologies in cardiac differentiation cultures (embryonic atrial-like, ventricular-like and nodal-like) [27]. To characterize the functional maturity of cardiomyocytes, action potential characteristics were measured in a single-cell level by whole-cell patch clamp. Nodal-like, atrial-like and ventricular-like cardiomyocytes were detected based on action potential amplitude (APA), action potential duration at the 90% of repolarization (APD_90_), maximum diastolic potential (MDP) and maximum upstroke velocity (maximum rate of rise of the action potential stroke, dV/dt_max_) (Fig. 2, Table 1). Quantification of all three different cardiomyocyte types for Bmp4 treated cardiomyocytes revealed similar representation of each cardiomyocyte type to that in control cultures (Table 1). No significant differences of AP, MDP and APA were detected between Bmp4 treated and vehicle-treated ventricular-like cardiomyocytes, whereas, lower upstroke dV/dt_max_ was measured for Bmp4-treated sample compared to control (p<0.01, Table 2). Although the action potentials of Bmp4 induced cardiomyocytes are mostly similar to untreated cardiomyocytes, Bmp4 induction appears to slightly alter the action potential of ventricular cardiomyocytes. These data suggest that the Bmp4 induced cardiomyocytes from Isl1-CPCs have typical characteristics of action potential parameters of untreated cardiomyocytes.

**Table 1:** Comparison of cardiomyocyte (CM) types from mESC-derived Isl1-CPCs in the absence or presence of Bmp4 treatment. The number of cardiomyocytes (# of CM) and the proportion of cardiomyocytes (% of total) exhibiting nodal-, atrial- and ventricular-like properties are shown (n = 4).

	Nodal-like CM		Atrial-like CM		Ventricular-like CM		Total CM
	# of CM	% of total	# of CM	% of total	# of CM	% of total	
Control	8	31	9	35	9	35	26
Bmp4	8	28	8	28	13	45	29

**Table 2:** Comparison of action potential parameters for Isl1-CPC derived cardiomyocyte cultures in the absence or presence of Bmp4 treatment (n = 4).

	APA (mV)	MDP (mV)	*dV/dt_max_*(mV/ms)	APD90 (ms)
Control	75.72±12.8	−51.34±12.1	18.38±3.2	227.12±45.5
Bmp4	69.03±8.8	−60.2±6.6	10.75±2.6	265.36±121.1

### Isl1-CPC derived cardiomyocytes exhibit periodic Ca^2+^ transients

**Figure 3:**
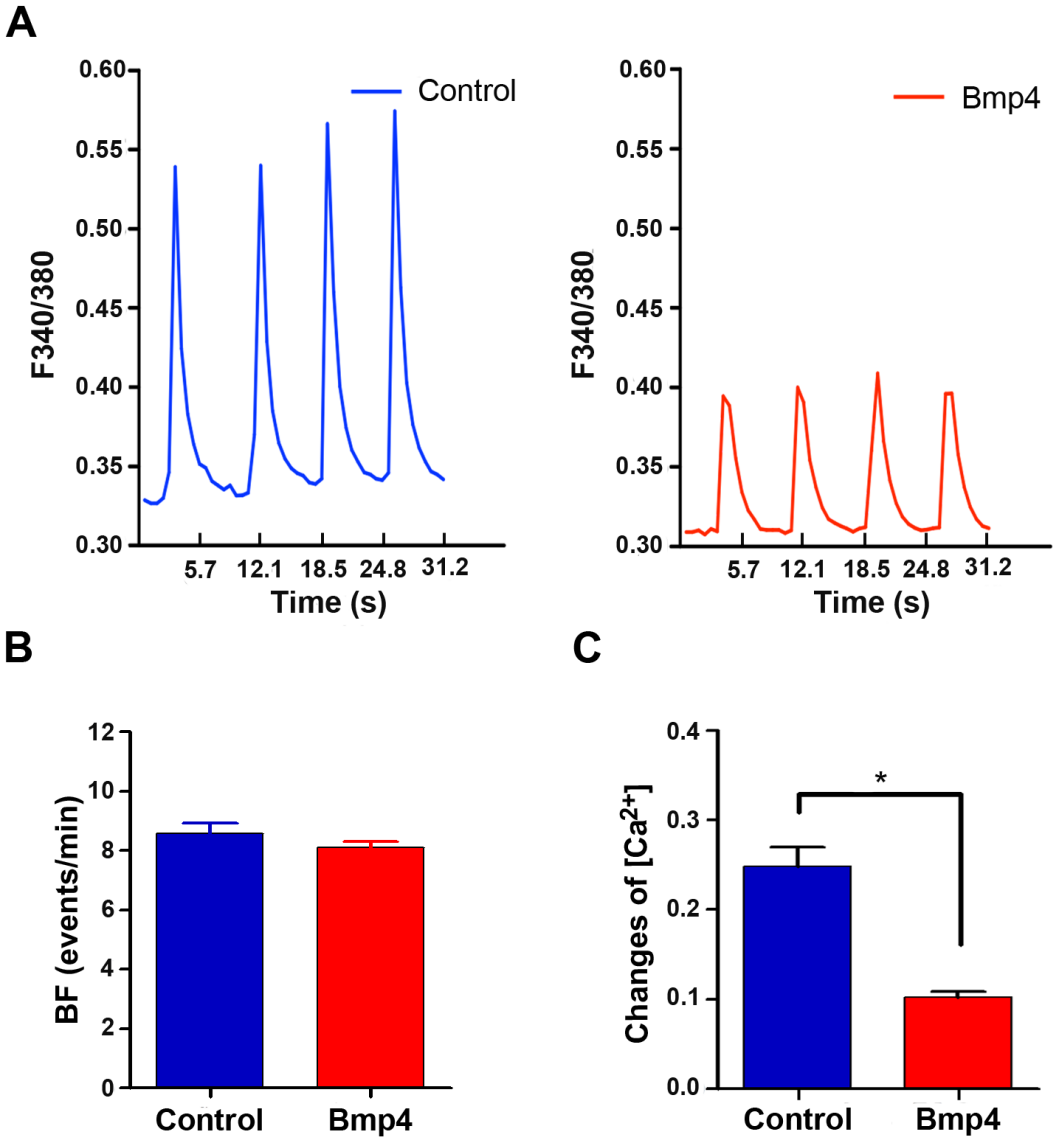
Calcium transients of cardiomyocytes derived from Isl1-CPC after cell loading with Ca^2+^ indicator fura-2. (A) Representative recordings for increase in [Ca^2+^]i transient amplitude and frequency was measured following loading of control and Bmp4-treated cardiomyocyte cultures with Ca^2+^ indicator fura-2. Cytosolic Ca^2+^ of spontaneous beating cardiomyocyte was measured by ratio of fluorescence intensity at 340 nm and 380 nm (F340/F380). The measurements were recorded 6 days of incubation after Isl1-CPC isolation. (B) The quantitative representation of beating frequency (BF) of calcium transients per minute is shown by bar graphs (n = 3). Experiments were repeated 3 times and 10–15 cells were measured in each experiment. (C) Change of [Ca^2+^] was calculated from Ca^2+^ transient recordings for cardiomyocyte cultures differentiated in the absence or presence of Bmp4. *p<0.05; n = 3 independent experiments.

The contraction and relaxation of cardiomyocytes are regulated by the cyclic changes in cytosolic Ca^2+^ concentrations, as measured by Ca^2+^ transients. Measuring calcium influx is a reliable indicator of functionality of cardiomyocytes. Therefore, we measured spontaneous intracellular calcium influx of Isl1-CPC-derived cardiomyocytes treated with Bmp4 using Fura-2 acetoxymethyl ester, a calcium-sensitive dye. We found that Isl1-CPC-derived cardiomyocytes showed spontaneous and rhythmic Ca^+2^ transients and similar frequency in Bmp4 treated and control cardiomyocytes (Fig. 3A and 3B; S5 Fig.). Interestingly, Bmp4 induced cardiomyocytes appeared to exhibit lower amplitude changes for Ca^2+^ concentration compared to control, suggesting that Bmp4 derived cardiomyocytes might be at an early stage of development with lower amplitude of calcium transients (Fig. 3A and 3C; S5 Fig.).

### TBX family transcription factors, Tbx 5 and Tbx 20, are downstream targets of Bmp4-induced cardiomyocyte differentiation of Isl1-CPC

**Figure 4:**
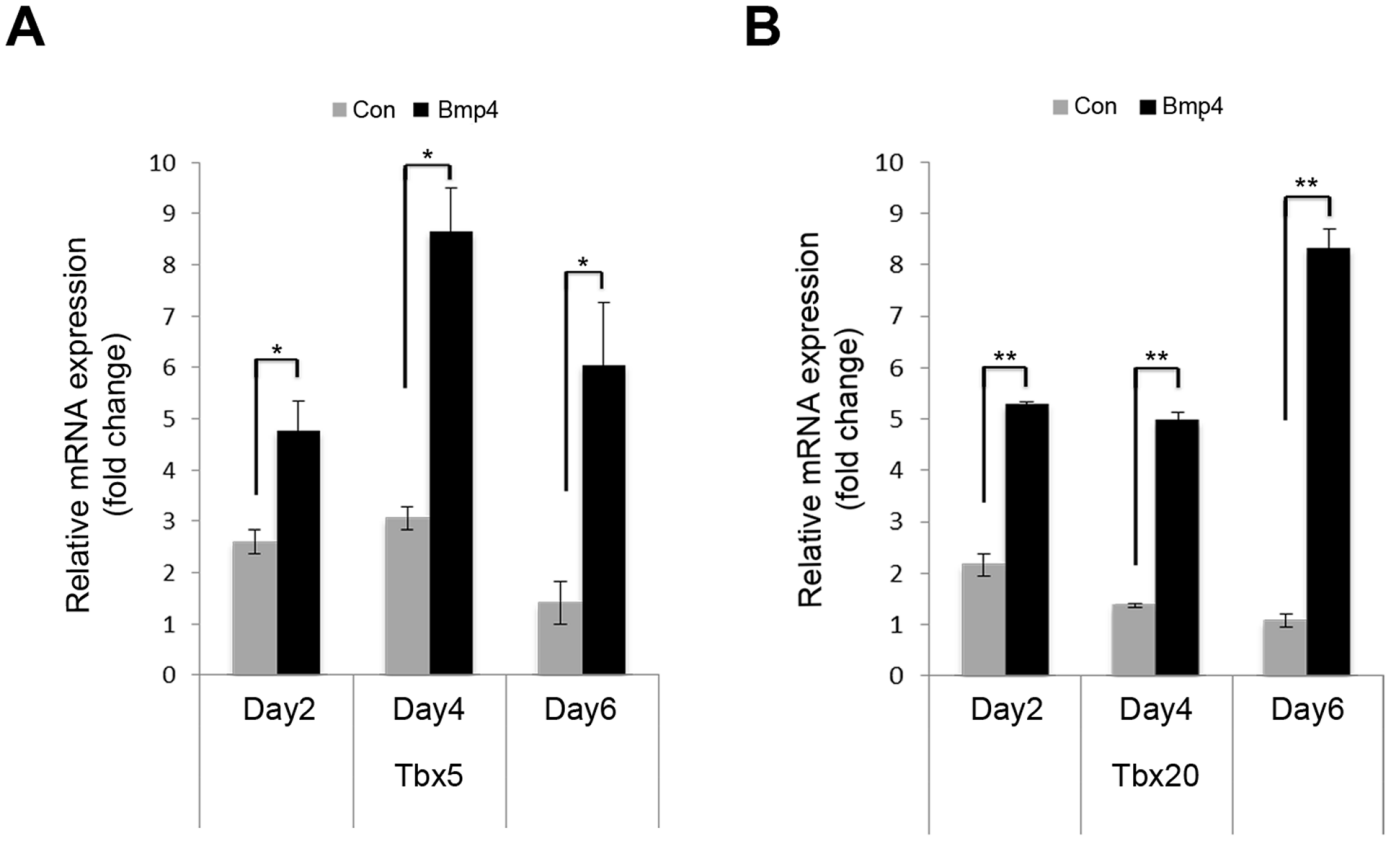
Bmp4 signaling enhances transcription factor *Tbx5* and *Tbx20* mRNA expression. FACS-sorted GFP+ Isl1-CPCs were cultured in the presence of Bmp4 or vehicle control. mRNA was collected following 2, 4 and 6 days of Bmp4 treatment. qRT-PCR analysis of duplicates from three independent experiments for *tbx5* (A) and *tbx20* (B) expression is normalized by housekeeping gene, Gapdh. *p<0.05; **p<0.01; n = 3 independent experiments.

To explore how Bmp4 enhances cardiac differentiation, mRNA analysis of Bmp4 treated and untreated purified Isl1-CPC was performed for early cardiac differentiation markers at day 2, 4 and 6 of differentiation. Our analysis showed a significant increase in T-box transcription factors (Tbx) Tbx 5 and Tbx 20 mRNA expression in cultures treated with Bmp4 compared to untreated control (Fig. 4). This finding is consistent with previous studies that show the essential cardiogenic roles played by Tbx 5 and Tbx 20 during heart development and suggests that Bmp4-mediated induction of Tbx 5 and Tbx 20 gene expression may account for the enhanced cardiac differentiation in Bmp4 treated Isl1-CPC cultures [28]–[30].

## Discussion

In order to fully harness the potential of cardiac regenerative medicine, it will be important to utilize a population of cardiac progenitor cells that is derived from a renewable source with high cardiac differentiation potential. Isl1-CPCs derived from pluripotent stem cells are an attractive source for cardiac cellular therapy due to its authentic cardiac origin and ability to generate an unlimited number of cells, sufficient for regeneration of damaged tissue [10], [12]–[14]. Although Isl1-CPCs have intrinsic cardiogenic potential, it is necessary to improve the cardiac differentiation efficiency in order to generate scalable quantities of cardiovascular cells for potential tissue regeneration. In this study, we aimed to improve cardiac differentiation of Isl1-CPCs by investigating the role of inducing Bmp4 upon a highly enriched population of Isl1-CPCs isolated from genetically modified mouse embryonic stem cells that allow for expression of GFP upon activation of the *mef2c* gene that is specifically in the Isl1 domain of the anterior heart field.

In our study, we showed that mRNA expression of cardiac progenitor marker genes *Isl1*, *Gata4* and *Nkx2.5* were significantly enriched in GFP+ progenitors (S1B Fig.). We also observed that these GFP+ cells represented a highly enriched population of CPCs via double staining for Isl1 and lineage markers (S1C-E Fig.). Consistent with the previous studies by Bu et al. and Musunuru et al., a small percentage of these CPCs co-expressed Isl1 and lineage markers, suggesting that they may represent lineage-committed, unipotent progenitors [13], [26]. It is interesting to note that virtually all cTnT (green) positive cells (97.2±2.8%) co-expressed SMA (red) in CPCs doubly stained for these markers (S1E Fig.). This suggests that Isl1+/cTnT+ cells are also SMA+ and represent a subpopulation of Isl1+/SMA+ cells. Since Isl1+/SMA+ and Isl1+/CD31+ lineage-restricted progenitors represented 15.9% and 0.3% of total Isl1-CPCs, respectively (S1D Fig.), our GFP+ cells thus represented a highly enriched population of uncommitted CPCs (83.8%). Additionally, SMA may be an early marker during cardiac differentiation of CPCs *in vitro*, consistent with the previous studies in which SMA was shown to be expressed in ESC-derived cardiomyocytes and the embryonic hearts [31], [32].

We further characterize specifically cardiac progenitor-derived cardiomyocytes and demonstrate the direct and inductive role of Bmp4 on Isl1 cardiac progenitors in order to increase the efficiency of cardiac differentiation. Bone morphogenetic proteins (Bmps) have been implicated in promoting cardiac differentiation both *in vivo* and *in vitro* settings. Bmp4 is expressed during heart muscle development, and Bmp4 conditional mutant mice display delayed myocardial differentiation [33]. Bmps have also been suggested to promote cardiac differentiation in embryonic stem cell cultures [3], [22], [34]. Until this study, the role of Bmp4 in Isl1 expressing cardiac progenitors restricted to the anterior heart field has not yet been studied. We show that induction of Bmp4 during differentiation of Isl1-CPCs leads to a significant increase of cTnT+ terminally differentiated cardiomyocytes evidenced by the appearance of sarcomeric striated structure and beating foci (Fig. 1 and S3 and S4 Figs.). This study is the first to show that Bmp4 induces cardiomyogenesis in a pure/homogenous population of cardiac progenitors. Enhancing the cardiac differentiation potential of Isl1-CPCs using Bmp4 treatment could further make it an attractive source for cardiac cell therapy.

Upon induction of differentiation, cardiomyocytes generated from Isl1-CPCs exhibit proper electrophysiological properties such as action potentials (Fig. 2, Table 1 and 2) and Ca^2+^ transients (Fig. 3). Nodal-like, atrial-like and ventricular-like cardiomyocytes were detected and exhibited proper action potential amplitude (APA), action potential duration (APD), maximum diastolic potential (MDP) and maximum upstroke velocity. With induction of Bmp4, there were no appreciable differences in the measured parameters except lower upstroke dV/dt_max_ for the Bmp4 treated cardiomyocytes. Interestingly, Bmp4 induced cardiomyocytes also appeared to exhibit lower amplitude changes for Ca^2+^ concentration compared to control (Fig. 3). It is possible that Bmp4 expression may influence the activities or composition of the channels involved in regulating action potential and calcium transient. We have performed real-time quantitative reverse transcription PCR (RT-qPCR) for the expression of alpha 1c and 1d subunit of the L-type calcium channel (Cacna1c or 1d), sodium/calcium exchanger (Ncx1), ryanodine receptor (Ryr2) and sarcoplasmic/endoplasmic reticulum calcium ATPase 2 (Serca2) (S6 Fig.). There appeared to be a modest increase in the expression of Cacna1c and Ncx1 while there were no significant differences in the expression of Cacna1d, Ryr2 and Serca2 in Bmp4-treated culture compared with control. Thus, it is less likely that Bmp4 regulates calcium transients mainly by altering calcium channel expression, and additional mechanism(s) may account for the decreased calcium transients in Bmp4-treated culture. For example, it has been suggested that Bmp4 is able to decrease electrical activity in developing neurons by enhancing p38 MAPK-mediated negative modulation of voltage-gated Na^+^ and Ca^2+^ channel function [35]. Additionally, Bmp2 has been implicated in reducing the number of voltage-gated Ca^2+^ channel in developing mice myotubes [36]. Future work will be warranted to explore whether Bmp4 may decrease electrical activity of Isl1-CPCs derived cardiomyocytes by activation of p38 signaling pathway-mediated inhibition of voltage-gated Na^+^ and Ca^2+^ channel function and/or decreasing the number of voltage-dependent Na^+^ and Ca^2+^ channels.

The members of the T-box gene family encode for transcription factors that are characterized by a highly conserved DNA-binding region, which recognizes a specific DNA element, the T-half site, thereby mediating transcriptional activation or repression [30]. The Tbx family of transcription factors is known to be important in regulating myocardial proliferation and patterning. For example, after establishing the cardiac tube in the late gastrulation stage during embryonic development, the elongation and formation of chambers is accompanied by myocardial patterning, which T-box genes Tbx5 and Tbx20 play crucial roles [30]. Tbx20 is also expressed during the first heart field and secondary heart field throughout cardiac development. Although Tbx5 and Tbx20 have important roles during cardiac development, the factors that control their expression are not fully understood. In our study, Tbx5 and Tbx20 show increased expression upon induction of Bmp4 and are likely to be downstream targets of Bmp4 in Isl1-CPC differentiation (Fig. 4). Future comprehensive gene screening can be performed to identify downstream genes regulated by Bmp4 in Isl1-CPCs, which may provide key information for the cardiac differentiation program in Isl1-CPC. Collectively these findings suggest a cardiomyogenic role for Bmp4 directly on purified Isl1 cardiac progenitors.

Adult progenitors and their differentiated progeny could lead to the development of novel regenerative strategies for patients who suffer from heart disease. However, ESC-derived CPCs could solve the age related decline in CPC function. The heart has a limited potential to repair itself upon injury through cardiomyocyte renewal [37]–[39] and/or stem cell activation [40], [41]. However, this limited regenerative potential declines by aging as resident adult CPCs age and lose some of their regenerative potential in adulthood. ESC- or iPSC-derived CPCs could provide a solution to this age related decline in function, as stem cell derived CPCs and cardiomyocytes exhibit embryonic-like and less mature phenotypes. A general concern about using ESC/iPSC and their derivatives is the potential risk of teratoma formation and genomic alternations. However, we anticipate minimal risk of teratoma formation of Isl1-CPCs since they are enriched by FACS sorting and are highly committed cardiovascular cells. Additionally, certain small molecules could be used to selectively eliminate ESCs or iPSCs in differentiated cultures [42], [43]. By using early passage ESC or iPSC lines coupled with close screening of genomic integrity, ESC- or iPSC-derived Isl1-CPCs would provide a valid cell source for cell-based therapies for cardiac repair.

As Isl1-CPCs are an attractive source for cellular therapy, using a purified population of cardiac Isl1-CPCs is important for higher efficiency of cardiac differentiation and for further understanding the mechanisms behind efficient cardiac differentiation. There has been *in vivo* characterization of cardiac progenitors expressing Isl-Cre based strategy. Several laboratories have isolated and characterized Isl1-CPCs from differentiation of embryonic stem cells (ESCs) [10], [12]–[14]. For the isolation and fate-mapping of *Isl1+* cardiovascular progenitor cells, the Cre recombinase gene controlled by the *Isl1* promoter and a Dsred or yellow fluorescent protein (YFP) reporter was used [12], [13], [18]. However, since Isl1 is also expressed in motor neurons and pancreatic islet cells, the Isl1-Cre transgenic reporter system may select both neural crest and secondary heart field originated cells [17], which the former complicates analysis. Therefore, the AHF-GFP transgenic system, which enables isolation of GFP+ Isl1 expressing cells, is superior to Isl1-Cre in that, the Mef2c promoter driving GFP contains not only Isl1 binding sites but also Gata4, another cardiac progenitor marker, in order to specifically select cardiac progenitors. Furthermore, the GFP+ Isl1 cells from the AHF-GFP transgenic can differentiate into cardiomyocytes, smooth muscle cells, and endothelial cells and exhibit proper electrophysiological and functional properties of differentiated cells. Future efforts will be made to investigate whether AHF-GFP+ Isl1 CPCs can repair and regenerate injured hearts and whether Bmp4 can enhance cardiac muscle formation after implantation of Isl1 CPCs in animal models.

## Conclusion

Heart failure remains as one of the leading causes of mortality in the developed world. Cell-based therapy to regenerate damaged cardiac tissue is an attractive strategy to treat heart disease. As there may be limitations for adult cardiac progenitor cells for cell therapy regarding tissue accessibility and proliferative potential, embryonic stem cell derived cardiac progenitor cells may serve as a source of cells with unlimited proliferative potential and differentiation capacity. Embryonic stem cell derived Isl1-CPCs may serve as a valuable source of cells due to its 1) authentic cardiac origin and 2) unlimited generation of cells upon derivation from pluripotent stem cells. Here we characterize and investigate the role of Bmp4 on enhancing the cardiac differentiation potential of Isl1-CPCs. We show that Bmp4 enhances differentiation of functional cardiomyocytes showing rhythmic action potential and calcium transient from a pure population of Isl1-CPCs that are isolated from transgenic AHF-GFP expressing embryonic stem cells. Future studies may be directed to test the cardiac differentiation potential of Isl1-CPCs *in vivo* and whether supplementing with Bmp4 could promote enhanced muscle formation *in vivo* for cardiac repair. Additionally, ongoing efforts are made to isolate mesodermal ISL1 CPCs from human ESCs and iPSCs equivalent to AHF-GFP, Isl1 CPCs from murine ESCs. The current study has been focused on studying the role of Bmp4 in murine Isl1-CPCs, future research will be performed to test the effect of BMP signaling on cardiac differentiation of human ISL1-CPCs *in vitro* and *in vivo*. Although many challenges still remain, understanding Isl1-CPCs may lead to potential novel strategies in targeting heart regeneration and repair.

## Supporting Information

S1 Fig **Murine ESC-derived Isl1-CPC.** (A) FACS profile of EB day 5.5 differentiated AHF-GFP ES cells. (B) qRT-PCR analysis of *isl1*, *gata4* and *nkx2.5* expression normalized by Gapdh in FACS-sorted GFP^+^ and GFP^−^ cell populations. n = 3 independent experiments, *p<0.05. (C) Double staining for Isl1 and α-smooth muscle actin (SMA) (left panel), cardiac troponin T (cTnT) (middle panel) or CD31 (right panel). Staining was performed on cytospun samples after cell isolation by fluorescent activated cell sorting (FACS) from embryoid body day 5.5 (EB5.5) culture. Isl1 is presented in red and SMA, cTnT and CD31 in green. Scale bar 50 µm. (D) Quantification of double positive cells after staining for Isl1 and SMA, cTnT or CD31 as described in C. (E) Double staining for SMA (red) and cTnT (green) in Isl1-CPCs from EB5.5 culture. It appeared that nearly all cTnT (green) positive cells (97.2±2.8%) co-expressed SMA (red). Data represents 3 independent experiments with 6 fields quantified per experiment. Scale bar, 50 µm. Stainings presented in green (C, E) were originally performed with a far red fluorophore to distinguish the staining from the endogenous GFP used for FACS. Far red staining (cyan) was converted to green with Image J to discriminate it from nuclei staining (blue) on merged images.
(PDF)

S2 Fig **FACS-purified GFP+ Isl1-CPCs give rise to cardiomyocytes, smooth muscle cells and endothelial cells** ***in vitro*** **.** Isl1-CPCs were purified at day 5.5 of EB formation and cultured on 16-well chamber slides coated with fibronectin. After 6 days in culture, cells were fixed, immunostained with antibodies against cardiomyocyte-specific protein cTnT (A), smooth muscle-specific protein SMA (B) and endothelial cell-specific protein CD31 (C), and quantified (D). Antibody specific staining: red or green. Nuclei: blue (Hoechst). Scale bar: 100 µm.
(PDF)

S3 Fig **Isl1-CPCs differentiated into sarcomere-forming cardiomyocytes** ***in vitro*** **.** Isl1-CPCs were isolated by FACS on EB5.5 and plated on fibronectin-coated 8-well chamber slides. Cells were treated with vehicle control (A) or Bmp4 (25 ng/ml) (B) six hours after plating. Cells were fixed six days after plating and stained for cardiac troponin T (cTnT). Hoechst (blue), cTnT (green). Scale bar, 20 µm.
(PDF)

S4 Fig **Increased beating foci in Bmp4-treated Isl1-CPC-derived cardiomyocyte culture.** Isl1-CPCs were isolated by FACS on EB5.5 and plated on fibronectin-coated 384-well plates. Cells were treated with vehicle control or Bmp4 (25 ng/ml) six hours after plating. Three fields per treatment were quantified for beating foci six days after the initiation of differentiation. Data represented four independent experiments. *p<0.05.
(PDF)

S5 Fig **Images of calcium intake in the absence and presence of Bmp4.** Images of the measurement of spontaneous intracellular calcium influx of Isl1-CPC derived cardiomyocytes treated with or without Bmp4, using Fura-2 acetoxymethyl ester, a calcium-sensitive dye. (A,C) Maximum and minimum influx of Ca2+ in ICPCs without Bmp4 treatment (B, D) Maximum and minimum influx of Ca2+ in ICPCs with Bmp4 treatment.
(PDF)

S6 Fig **Gene expression of calcium channels.** Isl1-CPCs were isolated by FACS at EB5.5 and plated on fibronectin-coated 24 well plates. Cells were treated with vehicle control or Bmp4 (25 ng/ml) six hours after plating. RNA was extracted six days after plating and processed for Real-Time Quantitative Reverse Transcription PCR (RT-qPCR) for the expression of alpha 1c and 1d subunit of the L-type calcium channel (Cacna1c or 1d), sodium/calcium exchanger (Ncx1), ryanodine receptor (Ryr2) and sarcoplasmic/endoplasmic reticulum calcium ATPase 2 (Serca2). *p<0.05. Data represent three independent experiments, and qPCR was performed in triplicates using Gapdh as housekeeping gene.
(PDF)

S1 Movie **Beating cardiomyocytes derived from purified Isl1-CPCs treated with vehicle control.**
(AVI)

S2 Movie **Beating cardiomyocytes derived from purified Isl1-CPCs treated with Bmp4.**
(AVI)
