# Supplementary figures and images for: Functional Cardiomyocytes Derived from Isl1 Cardiac Progenitors via Bmp4 Stimulation
Source: PLoS One. 2014 Dec 18;9(12):e110752. doi: 10.1371/journal.pone.0110752 (PMC4270687; doi:10.1371/journal.pone.0110752)

**Figure S1**

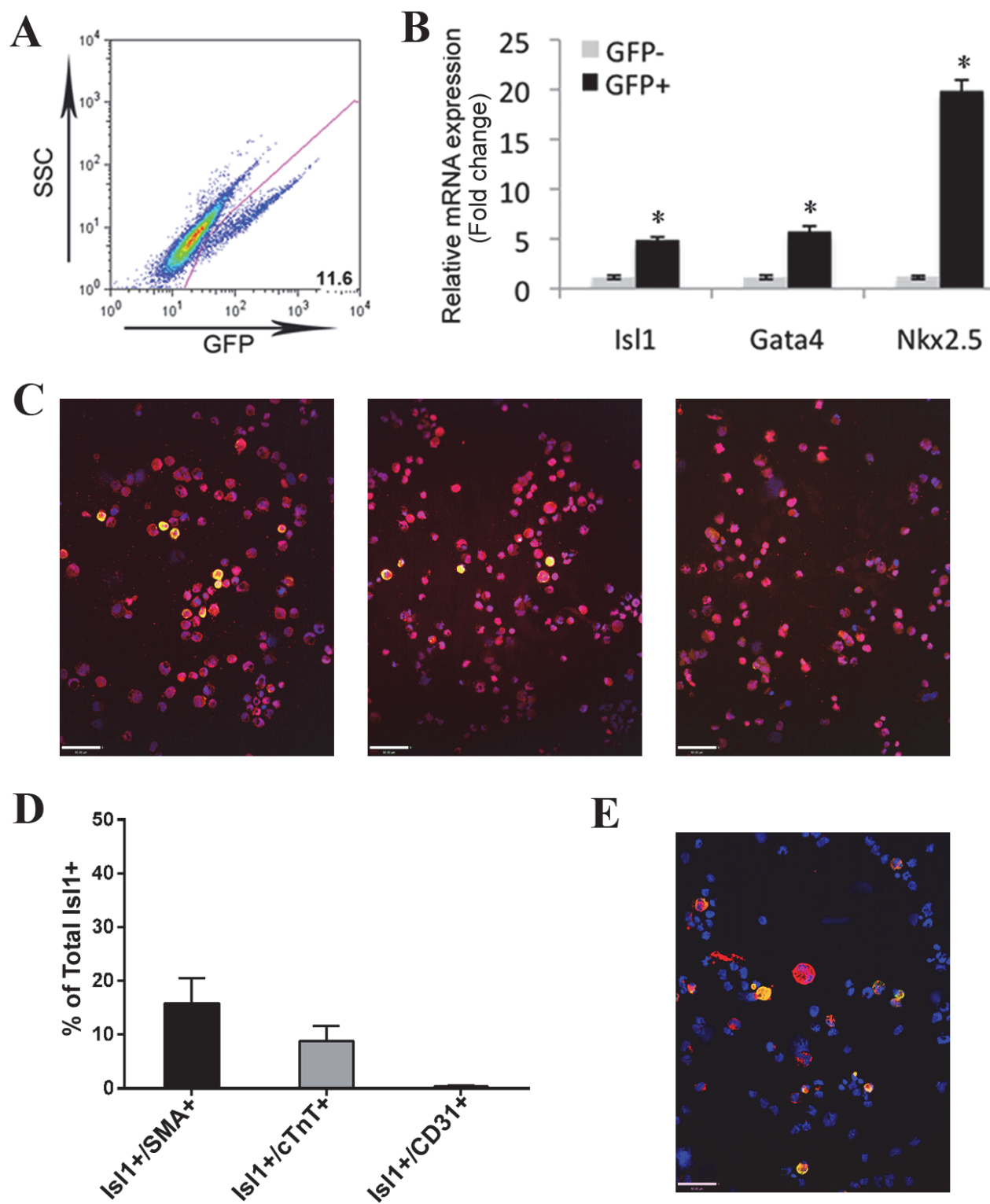

Supplement: S1 Fig — Murine ESC-derived Isl1-CPC. (A) FACS profile of EB day 5.5 differentiated AHF-GFP ES cells. (B) qRT-PCR analysis of isl1, gata4 and nkx2.5 expression normalized by Gapdh in FACS-sorted GFP+ and GFP− cell populations. n = 3 independent experiments, *p<0.05. (C) Double staining for Isl1 and α-smooth muscle actin (SMA) (left panel), cardiac troponin T (cTnT) (middle panel) or CD31 (right panel). Staining was performed on cytospun samples after cell isolation by fluorescent activated cell sorting (FACS) from embryoid body day 5.5 (EB5.5) culture. Isl1 is presented in red and SMA, cTnT and CD31 in green. Scale bar 50 µm. (D) Quantification of double positive cells after staining for Isl1 and SMA, cTnT or CD31 as described in C. (E) Double staining for SMA (red) and cTnT (green) in Isl1-CPCs from EB5.5 culture. It appeared that nearly all cTnT (green) positive cells (97.2±2.8%) co-expressed SMA (red). Data represents 3 independent experiments with 6 fields quantified per experiment. Scale bar, 50 µm. Stainings presented in green (C, E) were originally performed with a far red fluorophore to distinguish the staining from the endogenous GFP used for FACS. Far red staining (cyan) was converted to green with Image J to discriminate it from nuclei staining (blue) on merged images. (PDF) [file pone.0110752.s001.pdf]

Figure S2

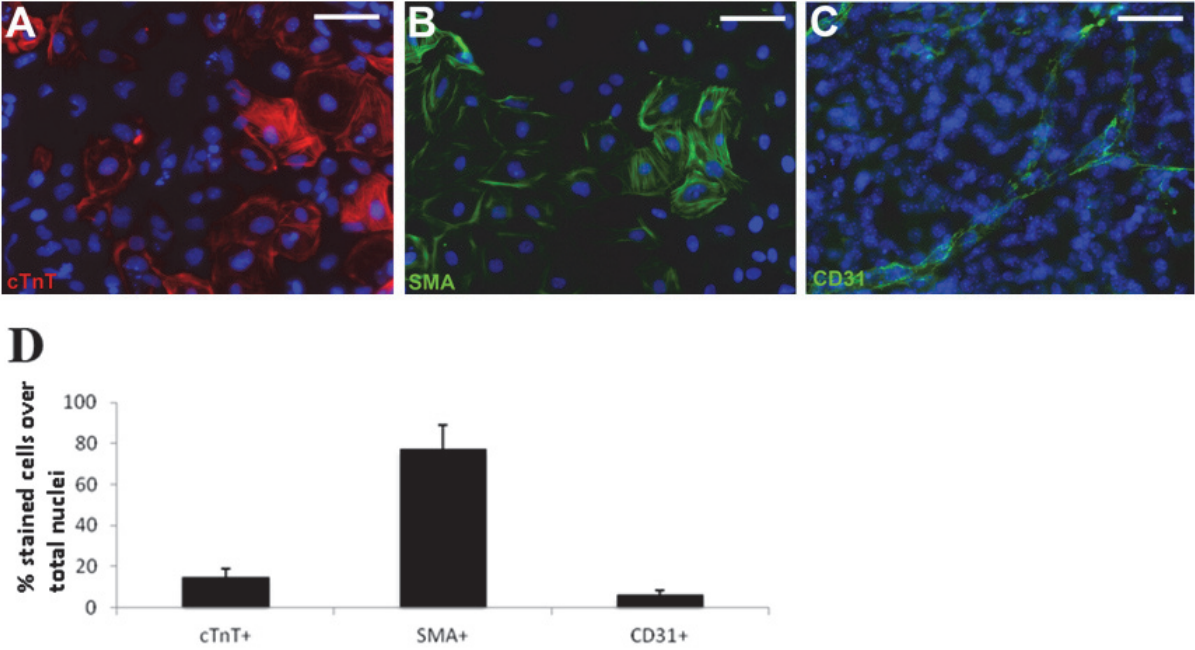

Supplement: S2 Fig — FACS-purified GFP+ Isl1-CPCs give rise to cardiomyocytes, smooth muscle cells and endothelial cells in vitro . Isl1-CPCs were purified at day 5.5 of EB formation and cultured on 16-well chamber slides coated with fibronectin. After 6 days in culture, cells were fixed, immunostained with antibodies against cardiomyocyte-specific protein cTnT (A), smooth muscle-specific protein SMA (B) and endothelial cell-specific protein CD31 (C), and quantified (D). Antibody specific staining: red or green. Nuclei: blue (Hoechst). Scale bar: 100 µm. (PDF) [file pone.0110752.s002.pdf]

Figure S3

A

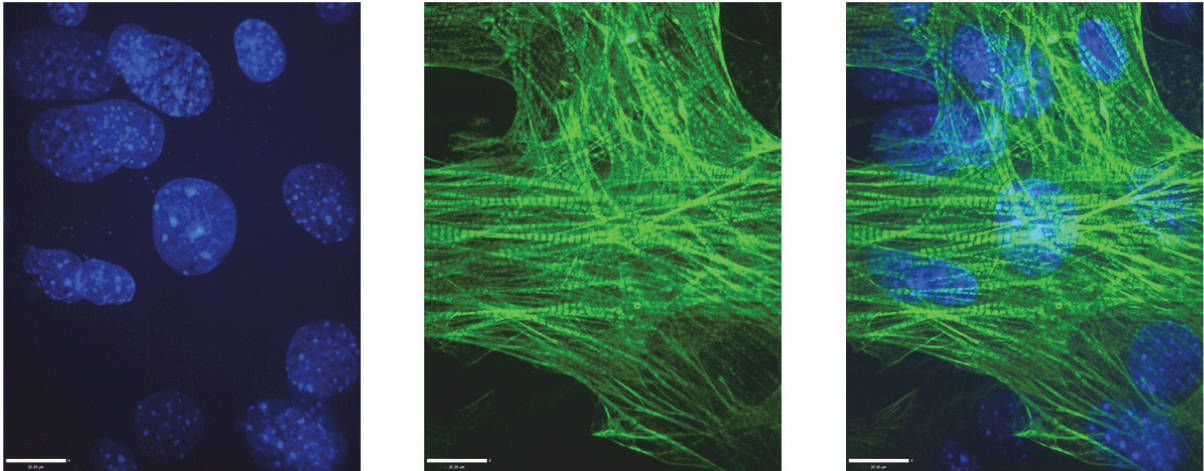

B

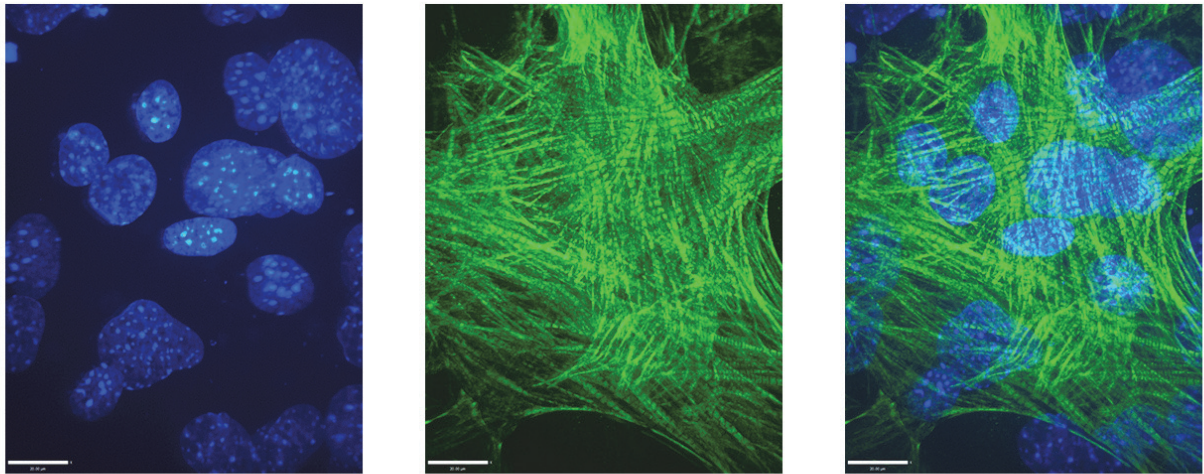

Supplement: S3 Fig — Isl1-CPCs differentiated into sarcomere-forming cardiomyocytes in vitro . Isl1-CPCs were isolated by FACS on EB5.5 and plated on fibronectin-coated 8-well chamber slides. Cells were treated with vehicle control (A) or Bmp4 (25 ng/ml) (B) six hours after plating. Cells were fixed six days after plating and stained for cardiac troponin T (cTnT). Hoechst (blue), cTnT (green). Scale bar, 20 µm. (PDF) [file pone.0110752.s003.pdf]

Figure S4

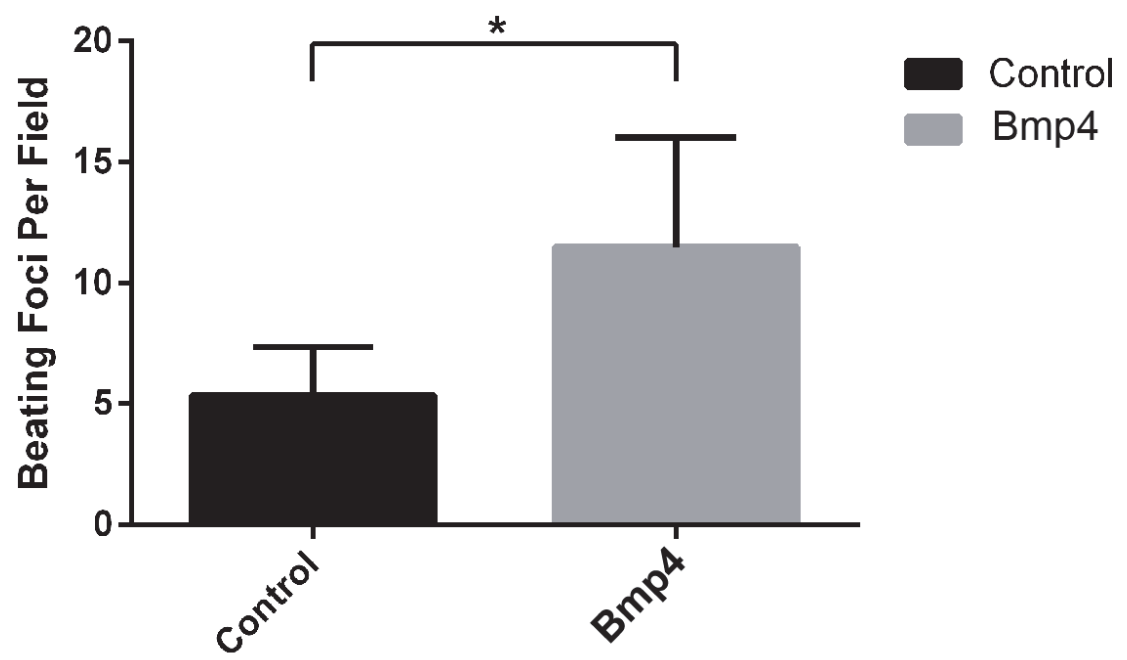

Supplement: S4 Fig — Increased beating foci in Bmp4-treated Isl1-CPC-derived cardiomyocyte culture. Isl1-CPCs were isolated by FACS on EB5.5 and plated on fibronectin-coated 384-well plates. Cells were treated with vehicle control or Bmp4 (25 ng/ml) six hours after plating. Three fields per treatment were quantified for beating foci six days after the initiation of differentiation. Data represented four independent experiments. *p<0.05. (PDF) [file pone.0110752.s004.pdf]

**Figure S5**

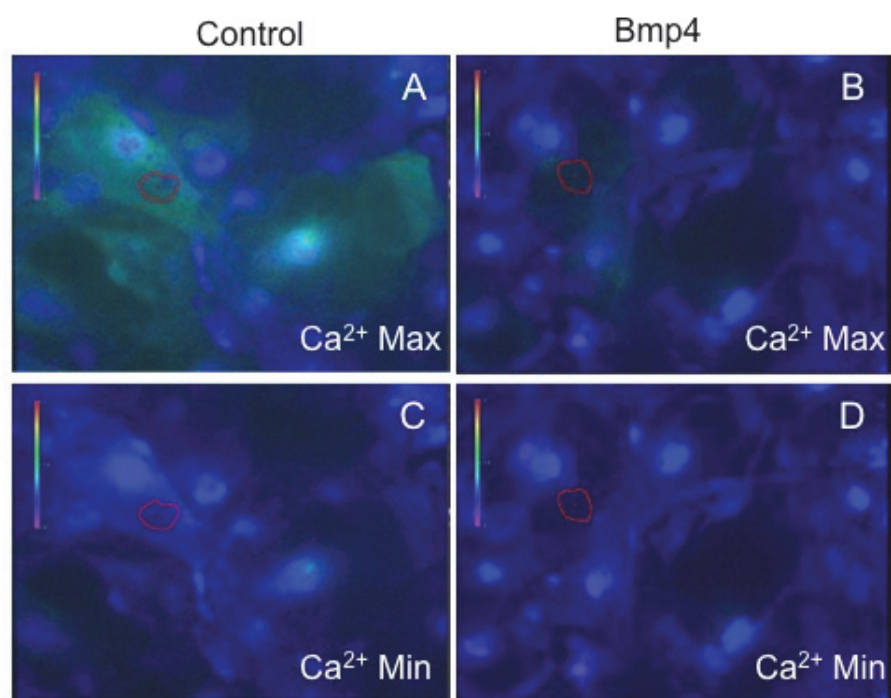

Supplement: S5 Fig — Images of calcium intake in the absence and presence of Bmp4. Images of the measurement of spontaneous intracellular calcium influx of Isl1-CPC derived cardiomyocytes treated with or without Bmp4, using Fura-2 acetoxymethyl ester, a calcium-sensitive dye. (A,C) Maximum and minimum influx of Ca2+ in ICPCs without Bmp4 treatment (B, D) Maximum and minimum influx of Ca2+ in ICPCs with Bmp4 treatment. (PDF) [file pone.0110752.s005.pdf]

Figure S6

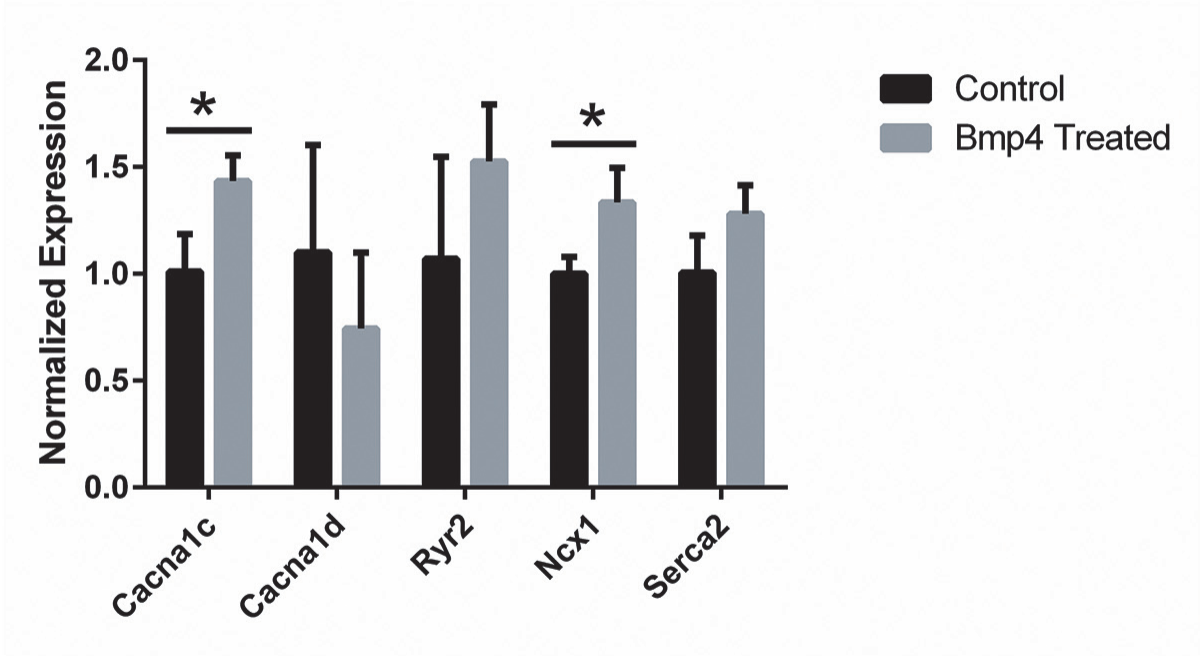

Supplement: S6 Fig — Gene expression of calcium channels. Isl1-CPCs were isolated by FACS at EB5.5 and plated on fibronectin-coated 24 well plates. Cells were treated with vehicle control or Bmp4 (25 ng/ml) six hours after plating. RNA was extracted six days after plating and processed for Real-Time Quantitative Reverse Transcription PCR (RT-qPCR) for the expression of alpha 1c and 1d subunit of the L-type calcium channel (Cacna1c or 1d), sodium/calcium exchanger (Ncx1), ryanodine receptor (Ryr2) and sarcoplasmic/endoplasmic reticulum calcium ATPase 2 (Serca2). *p<0.05. Data represent three independent experiments, and qPCR was performed in triplicates using Gapdh as housekeeping gene. (PDF) [file pone.0110752.s006.pdf]
